# Toothbrushing behavior changes among korean students pre- and post-COVID-19: lessons and policy implications

**DOI:** 10.1186/s12903-026-09051-6

**Published:** 2026-06-29

**Authors:** Ji-Young Son, Dong-Hun Han

**Affiliations:** 1 Department of Dental Hygiene, College of Bioecological Health, Shinhan University, Uijeongbu, Korea; 2 Department of Preventive and Social Dentistry, School of Dentistry, Seoul National University, Seoul, Korea (Republic of); 3 Dental Research Institute, Seoul National University, Seoul, Korea (Republic of)

**Keywords:** Covid-19, Health Behavior, Oral Health, School Health Services, Toothbrushing

## Abstract

**Background/Objectives:**

In South Korea, the nationally funded School Oral Health Room Program has promoted children’s oral hygiene practices since 1999; however, the COVID-19 pandemic disrupted these behaviors. This study investigates changes in toothbrushing practices among South Korean children aged 4–15 across before, during, and after Covid-19.

**Methods:**

We analyzed cross-sectional data from the Korean National Health and Nutrition Examination Survey (KNHANES VIII–IX, 2018–2023), including 5,251 children aged 4–15 years. Toothbrushing behaviors were assessed across three periods: Before COVID (2018–2019), during COVID (2020–2021), and after COVID (2022–2023). Complex-samples chi-square tests and logistic regression analyses (adjusted for sex and age) were conducted.

**Results:**

Brushing after lunch declined markedly across all age groups during and after the pandemic (e.g., 4–6-year-olds: 83.5% to 55.1%). In contrast, brushing before bedtime increased post-pandemic, particularly among older children (e.g., 10–12-year-olds: 59.6% to 74.2%). Brushing after breakfast declined during the pandemic with partial recovery, while brushing before breakfast showed mixed trends and after-dinner brushing decreased among older children.

**Conclusions:**

COVID-19 disrupted school-based toothbrushing, leading to reduced post-lunch brushing and increased bedtime brushing. These changes underscore the need for adaptable oral health programs that can sustain effectiveness during public health crises.

## Introduction

The World Health Organization (WHO) strongly recommends school-based oral health education and preventive strategies as key components of public health promotion [1]. In South Korea, the nationally funded School Oral Health Room Program has been implemented since 1999 to provide individualized oral health education and hands-on instruction in proper toothbrushing techniques for elementary, middle, and high school students. [2]. According to the Korean Dental Association, toothbrushing should be performed at least twice daily-particularly in the morning and before bedtime-while brushing after lunch is also recommended [3]. Furthermore, Korea’s Health Plan 2030 has set a national target of increasing the proportion of the population practicing post-lunch toothbrushing to 60% [4].

Since the establishment of the Oral Health Act in 2000, the Ministry of Health and Welfare has implemented various national oral health programs based on the periodic National Oral Health Survey [5]. As a result of these national initiatives, the prevalence of dental caries among children has steadily declined. In particular, the proportion of 5-year-olds with caries experience decreased from approximately 83% in 2000 to 62% in 2012, while the prevalence among 12-year-olds dropped from about 77% to 57% over the same period. These findings suggest that dental caries peaked around 2000 and has since followed an overall downward trend [6]. School-based oral health education, particularly toothbrushing instruction, not only improves oral health but also fosters lifelong self-care capabilities essential for maintaining oral hygiene [7, 8]. Since 2006, the post-lunch toothbrushing rate among middle and high school students has increased by an average of 0.9% annually [4]. In 2010, the prevalence of toothbrushing after lunch among adolescents (grades 7–12) was 38.6% of the total student population [9]. Over the past decade, the proportion of 12-year-old children practicing toothbrushing after lunch at school has shown a continuous upward trend [10]. However, among middle and high school students, the prevalence of post-lunch toothbrushing increased until 2011, after which it showed a slight decline.

The COVID-19 pandemic, however, disrupted children’s daily routines, including oral hygiene practices. While numerous studies have examined changes in general health behaviors such as diet, physical activity, and screen time [11–13], relatively little attention has been given to oral health. Similar patterns, including increased screen time and snack consumption, were observed among children and adolescents across Europe and Asia, while the United States showed comparatively smaller changes [14].

The impact of COVID-19 on health behaviors varied across countries according to public health measures. In the United States, relatively lenient and decentralized lockdown policies were adopted, whereas Italy enforced early and strict non-pharmaceutical interventions. The United Kingdom initially delayed lockdown and even discussed herd immunity before shifting to stricter measures, while China implemented a “Zero COVID” policy with mass testing and lockdowns. South Korea pursued rigorous containment through large-scale PCR testing, digital contact tracing, and transparent communication by the Korea Disease Control and Prevention Agency (KDCA) [15–18]. Although these measures were effective in reducing infection rates, they also revealed challenges in risk communication and continuity of daily health routines [19–22]. Despite extensive public health measures, evaluations of school-based oral health education and hygiene management remain limited. Evidence and guidelines on oral health behaviors-especially toothbrushing-during the pandemic are scarce, and oral health has received less attention than other health behaviors.

This study analyzes changes in children’s toothbrushing practices at home and school across before, during, and after pandemic periods, and examines the influence of national infection control policies on these behaviors. In doing so, it addresses a key gap in the literature, as few studies have investigated nationwide trends in oral hygiene behaviors during the COVID-19 pandemic. Using data from the Korea National Health and Nutrition Examination Survey (KNHANES), which includes health interviews, physical examinations, and oral examinations by trained dentists, this study evaluates shifts in school-based oral hygiene practices and the effectiveness of oral health education. The findings will provide evidence to improve collective toothbrushing programs and guide policy strategies to promote oral health among students, even during future public health crises.

## Methods

### Study population

Of the 43,745 participants of Korean National Health and Nutrition Examination Survey (KNHANES) VII, VIII and IX, 5,251 children between the ages of 4–15 years who reported having brushed their teeth were included. Participants with missing data on key variables (e.g., age, toothbrushing experience) or those outside the target age range were excluded. We used cross-sectional data from the National Health and Nutrition Examination Survey (KNHANES VIII and IX), an epidemiological survey conducted by the Korea Centers for Disease Control and Prevention (KCDC) between 2018 and 2023. The pre-COVID-19 period was defined using data from 2018–2019, the during-COVID-19 period using data from 2020–2021, and the post-COVID-19 period using data from 2022–2023. The KNHANES is a nationwide survey in Korea that began in 1998 and assesses various aspects of health, including dental health. The KNHANES employs a complex, two-stage stratified cluster sampling design to represent the non-institutionalized civilian population of Korea, using enumeration districts as the primary sampling units and households as the secondary sampling units. The sampling frame is further stratified across 17 provinces and metropolitan cities to ensure national representativeness. Since 2007, it has been conducted annually, with each year or combination of consecutive years constituting a nationally representative sample [23]. The survey uses a three-stage sampling process: primary sampling units (PSUs) of census blocks or resident registration addresses (~ 50–60 households) are selected, 20 households per PSU are chosen through field screening, and all household members aged ≥ 1 year are included. Approximately 10,000 individuals from 192 PSUs participate annually, with an overall target response rate of 75% [23]. The survey consists of a health interview and a comprehensive health examination carried out by trained medical professionals. In addition to age, demographic information such as sex, socioeconomic status, and education level is also collected. All participants provided informed consent prior to participation. For children, consent was obtained from parents or legal guardians, and the questionnaires were tailored by age group, with separate modules for children (10–11 years), adolescents (12–18 years), and adults (≥ 19 years) [24].

The original KNHANES data collection was approved by the Korea Disease Control and Prevention Agency (KDCA) Institutional Review Board (IRB No. 2018–01–03-P-A, 2018–01–03-C-A, 2018–01–03-2C-A, 2018–01–03-5C-A, 2018–01–03-4C-A, 2022–11–16-R-A), and all procedures followed the principles of the Declaration of Helsinki. The present study involved secondary analysis of existing de-identified data only; no additional IRB approval was required.

### Outcome variable and explanatory variable

Toothbrushing timing was assessed based on the question, "Did you brush your teeth yesterday?" Participants who answered "yes" were categorized into time. For our analysis, we utilized the variables for brushing before breakfast, after breakfast, after lunch, after dinner, and before bedtime. Age was categorized into preschoolers (ages 4–6), lower elementary school students (ages 7–9), upper elementary school students (ages 10–12), and middle school students (ages 13–15) because cognitive development and motivational structures vary by age [25]. Preschoolers tend to rely heavily on direct support from parents and teachers, younger elementary students are more influenced by external reinforcements such as praise and rewards, upper elementary students are more affected by peer influence and the formation of self-identity, and middle school students are more likely to understand the benefits of tooth-brushing and manage it autonomously [25].

### Statistical analysis

All variables were categorical, and descriptive characteristics of the study population were derived from sampling data, expressed as weighted percentages (%). Complex sample frequency analysis was used to examine tooth brushing timing by age group, and cross-tabulation analysis was conducted to identify trends across different years. General participant characteristics were identified using complex samples frequency analysis (Table 1). Sampling weights, strata, and PSU were specified. To compare the distribution of toothbrushing timing across age groups before COVID-19 (2018–2019), during COVID-19 (2020–2021), and after COVID-19 (2022–2023), complex-samples cross-tabulation analysis was applied. Results are presented as weighted percentages (%) with 95% confidence intervals (CIs). Using the complex samples chi-square test, the distribution of perceived oral health and toothbrushing timing across sociodemographic and health-related characteristics was examined. Statistical analyses were conducted using SPSS software (version 23.0; IBM Corp., Armonk, NY, USA). A P-value of < 0.05 was considered statistically significant. To compare toothbrushing frequency during and after COVID-19 with that before COVID-19 as the reference, complex-samples logistic regression analysis was performed. Adjusted odds ratios (aORs) were calculated after controlling for sex and age.

**Table 1:** Toothbrushing Frequency by Age and Period: Before, During, and After COVID-19

Age	Toothbrushing time	Before Covid-19 (2018–2019)		During Covid-19 (2020–2021)		OR_adj_^b^ (95% CI)	After Covid-19 (2022–2023)		OR_adj_^b^ (95% CI)	*P*-value^a^
		N	Weighted% (95% CI)	N	Weighted% (95% CI)		N	Weighted% (95% CI)		
4–6	Before breakfast	90	18.4 (16.2–20.8)	72	20.0 (14.9–26.3)	0.91 (0.62–1.32)	65	19.6 (15.2–24.9)	0.95 (0.81–1.12)	0.683
	After breakfast	301	61.2 (57.0–65.3)	192	52.9 (46.5–59.2)	1.41 (1.03–1.91)	163	60.0 (53.2–66.4)	1.06 (0.90–1.27)	0.027
	After lunch	406	83.5 (80.4–86.2)	209	59.4 (53.3–65.1)	3.53 (2.54–4.90)	158	55.1 (47.0–63.0)	4.08 (3.30–5.06)	< 0.001
	After dinner	172	34.1 (30.4–38.0)	129	36.0 (30.6–41.7)	0.92 (0.69–1.24)	97	35.3 (29.7–41.4)	0.96 (0.81–1.13)	0.716
	Before bedtime	320	67.0 (63.0–70.8)	245	67.3 (61.1–72.9)	0.99 (0.72–1.35)	199	69.5 (63.3–75.0)	0.88 (0.74–1.05)	0.607
7–9	Before breakfast	118	20.3 (17.5–23.4)	90	18.5 (14.8–23.0)	1.12 (0.81–1.54)	105	25.2 (20.2–31.0)	0.75 (0.63–0.89)	0.015
	After breakfast	383	66.7 (63.5–69.8)	292	66.3 (60.0–72.0)	1.02 (0.75–1.34)	261	62.8 (57.0–68.2)	1.18 (1.02–1.36)	0.297
	After lunch	303	51.5 (48.1–54.8)	176	37.8 (32.0–44.0)	1.74 (1.30–2.34)	113	26.3 (21.2–32.1)	2.93 (2.56–3.36)	< 0.001
	After dinner	231	39.0 (35.5–42.7)	159	34.8 (29.3–40.9)	1.19 (0.89–1.60)	147	34.3 (29.1–39.9)	1.22 (1.04–1.41)	0.191
	Before bedtime	340	60.6 (57.1–64.0)	315	69.9 (64.7–74.7)	0.67 (0.50–0.87)	295	71.5 (65.4–76.9)	0.62 (0.53–0.71)	< 0.001
10–12	Before breakfast	136	26.4 (22.9–30.3)	96	22.2 (17.3–28.0)	1.27 (0.88–1.82)	103	26.3 (21.6–31.6)	1.01 (0.83–1.22)	0.232
	After breakfast	308	61.3 (57.7–64.8)	251	55.3 (49.6–60.9)	1.27 (0.96–1.68)	247	61.9 (56.7–66.9)	0.98 (0.84–1.14)	0.049
	After lunch	225	44.9 (41.3–48.5)	171	40.3 (34.8–46.0)	1.21 (0.92–1.61)	92	22.2 (18.3–26.7)	2.86 (2.45–3.34)	< 0.001
	After dinner	215	41.7 (38.1–45.5)	157	34.9 (29.5–40.6)	1.34 (1.01–1.78)	129	32.6 (26.1–39.9)	1.48 (1.27–1.71)	0.007
	Before bedtime	288	59.6 (55.7–63.3)	301	69.4 (63.7–74.5)	0.65 (0.48–0.87)	297	74.2 (68.9–78.8)	0.52 (0.44–0.60)	< 0.001
13–15	Before breakfast	134	32.9 (29.3–36.6)	98	27.6 (22.1–33.9)	1.27 (0.90–1.79)	102	28.1 (23.1–33.7)	1.25 (1.05–1.48)	0.150
	After breakfast	228	57.9 (54.2–61.5)	176	48.3 (42.5–54.2)	1.47 (1.11–1.95)	192	53.6 (47.6–59.5)	119 (1.03–1.38)	0.011
	After lunch	169	46.5 (42.9–50.1)	136	36.7 (31.2–42.6)	1.48 (1.10–1.99)	103	27.8 (22.3–34.0)	2.28 (1.94–2.68)	< 0.001
	After dinner	196	48.7 (44.9–52.4)	142	39.9 (34.5–45.5)	1.42 (1.08–1.86)	137	39.6 (35.4–44.0)	1.44 (1.25–1.68)	0.003
	Before bedtime	229	56.6 (52.9–60.3)	232	66.7 (61.6–71.5)	0.65 (0.49–0.85)	247	67.2 (62.5–71.6)	0.64 (0.55–0.74)	< 0.001

Odds ratios are presented with 95% confidence intervals

The reference group was the period before COVID-19 (2018–2019), and in this analysis, the odds ratios represent the likelihood of not performing toothbrushing

Values are presented as the Unweighted number (N) (%)

Weighted percent, 95% confidence interval (CI), and P-value obtained by the chi-squared test

^a^P-values indicate differences in the distribution of toothbrushing times across the three survey periods (before, during, and after COVID-19)

^b^Age-and gender-adjusted odds ratio (OR_adj_) of toothbrushing times

## Results

### Toothbrushing frequency by age and period: before, during, and after COVID-19

Brushing after lunch showed a consistent decline across all age groups, with a sharp decrease during the pandemic and further reduction post-pandemic. Among 4–6-year-olds, brushing after lunch dropped from 83.5% before COVID-19 to 59.4% during and 55.1% after. In the 7–9 age group, it declined from 51.5% before to 37.8% during and 26.3% after. Similarly, in the 10–12 and 13–15 age groups, brushing after lunch significantly decreased from 44.9% and 46.5% before COVID-19 to 22.2% and 27.8% after, respectively. This decline was statistically significant across all age groups.

Brushing before bedtime increased across all age groups, particularly after COVID-19. In 7–9-year-olds, it rose from 60.6% to 71.5%, in 10–12-year-olds from 59.6% to 74.2%, and in 13–15-year-olds from 56.6% to 67.2%, showing a statistically significant increase. In the 4–6 age group, brushing before bedtime increased from 67.0% before COVID-19 to 69.5% after, though this change was not statistically significant.

Brushing after breakfast showed a fluctuating pattern, with a decline during the pandemic but a partial recovery post-pandemic in most groups. The 4–6 age group experienced a significant decrease during the pandemic but returned close to pre-pandemic levels afterward. Similarly, the 10–12 and 13–15 age groups showed a decrease during the pandemic, followed by recovery. However, the 7–9 age group did not show a statistically significant change.

Brushing after dinner showed a slight but consistent decrease among older children, particularly in the 10–12 and 13–15 age groups, where the decline was statistically significant. In the 4–6 age group, brushing after dinner decreased from 61.2% before to 52.9% during the pandemic but rebounded to 60.0% after. However, in the 10–12 group, it declined from 41.7% before to 32.6% after COVID-19, and in the 13–15 group, it dropped from 48.7% before to 39.6% after.

Brushing before breakfast exhibited mixed trends. In the 7–9 age group showed a significant post-pandemic increase, rising from 20.3% before COVID-19 to 18.5% during, and 25.2% after. However, in the 4–6, 10–12, and 13–15 age groups, the changes were not statistically significant.

## Discussion

This study examined changes in oral health behaviors among children and adolescents before and after the COVID-19 pandemic. The results revealed a continuous decline in post-lunch toothbrushing during and after the pandemic compared to the pre-pandemic period. In contrast, toothbrushing before bedtime— a representative oral hygiene behavior performed at home—showed an increasing trend, with the frequency demonstrating a notable upward trajectory over time.

In South Korea, strict social distancing and public health measures were implemented, including mandatory handwashing and mask-wearing. Noncompliance could result in both social blame and legal penalties [18]. When confirmed cases occurred, detailed information on individuals’ movements was made public to enable rapid tracing—an approach effective for containment but controversial in terms of privacy [18]. Although these containment measures influenced the development of hygiene habits among students, policy-level considerations regarding toothbrushing practices within schools were relatively insufficient. Due to concerns about infection risks in shared spaces, some schools restricted students from brushing their teeth in public restrooms or suspended group toothbrushing programs altogether [26, 27]. Such measures likely had a significant impact on students' oral health management and may partially explain the observed decline in post-lunch toothbrushing frequency during and after the pandemic.

The United Kingdom continued its programs under reinforced infection control guidelines, such as handwashing before and after brushing, individualized toothbrush storage, and socially distanced environments [28]. This example illustrates how national policies can determine the continuity of school oral health programs and emphasizes the need for policy-level strategies to protect oral health in future outbreaks.

The decline in post-lunch toothbrushing may be attributed to several factors. First, school-based toothbrushing was restricted as part of infection prevention, with many institutions prohibiting the practice when classes resumed [26, 27]. These restrictions, together with reduced access to dental care, adversely affected students’ oral health and widened disparities [29]. The suspension of local oral health programs also raised concerns about vulnerable groups [29]. Second, while guidelines emphasized handwashing and mask-wearing, no specific policies for school toothbrushing were provided [30], leaving schools to suspend the practice independently. Third, limited teacher awareness and engagement further hindered implementation, consistent with prior reports identifying teacher interest as a key barrier [31].

In contrast, bedtime toothbrushing increased at home, likely due to heightened parental supervision. This study found notable rises across all age groups—for example, from 59.6% to 74.2% among 10–12-year-olds. These trends suggest that increased time at home strengthened parental involvement, highlighting the complementary roles of schools and families and the need for coordinated, sustainable policies to safeguard oral health during future public health crises. Building on this finding, greater emphasis should be placed on parental and community involvement, which can complement school-based programs and provide a more comprehensive framework for children’s oral health.

As infection control policies prioritized droplet prevention, school toothbrushing practices were increasingly regarded as potential sources of aerosol spread. Consequently, students were discouraged from brushing at school, and some institutions closed designated areas. These measures, though intended to improve hygiene, paradoxically undermined infection control goals. Evidence shows that oral hygiene plays a key role in infectious disease prevention: professional plaque control can reduce influenza risk by up to 90% [32], chlorhexidine mouthwash lowers the incidence of ventilator-associated pneumonia [33], and poor oral hygiene has been linked to greater COVID-19 susceptibility [34]. Moreover, mouth rinses containing povidone-iodine (PI) or cetylpyridinium chloride (CPC) may help suppress coronavirus transmission [35]. Thus, oral hygiene management should be recognized not only for preventing dental caries but also as a critical public health measure in infection control.

During the COVID-19 pandemic, the South Korean government prioritized infectious disease control, while oral health behaviors received little policy attention. Consequently, school-based toothbrushing habits declined and have yet to return to pre-pandemic levels. The School Oral Health Room Program, introduced in 1999, has shown minimal growth, with only 417 schools (4.7%) establishing oral health rooms by 2015 [36]. Oral hygiene, however, is critical not only for preventing dental caries but also for reducing infectious disease risks [32]. Future pandemic responses should therefore emphasize toothbrushing as a core health behavior, alongside handwashing and mask-wearing, supported by stronger policy commitment.

To date, official reports on national toothbrushing policies, particularly concerning post-lunch toothbrushing in schools, remain scarce, with notable exceptions being South Korea and the United Kingdom [4, 37]. Although no official national reports have been documented in many developing countries, several studies have emphasized the importance of post-lunch toothbrushing. In Thailand, 92.9% of surveyed schools reported implementing after-lunch toothbrushing activities [38], while in Myanmar, some schools have already introduced post-lunch toothbrushing and highlighted the need for nationwide expansion [39].

It is therefore necessary to evaluate how toothbrushing-related policies during the COVID-19 pandemic have influenced children’s oral hygiene behaviors in the aftermath. Moreover, efforts should be made to collect and disseminate information on oral health and oral health behaviors across countries, and to establish a network-based platform that enables knowledge-sharing among schools. Such an information infrastructure could serve as a critical foundation for timely, evidence-based policy decisions in future public health emergencies.

This study has several limitations. First, the KNHANES relied on self-administered questionnaires, which may have overestimated toothbrushing behavior. For instance, while the Korea Youth Risk Behavior Survey reported a decline in post-lunch toothbrushing among adolescents (41.3% in 2011 to 38.5% in 2019), our KNHANES-based rates before COVID-19 were considerably higher (e.g., 67.0% in ages 4–6) [40], suggesting potential reporting bias. Future research should therefore incorporate observational methods to improve accuracy. Second, the KNHANES contains only one item on toothbrushing, limiting comparisons with other hygiene behaviors and preventing identification of specific barriers. In-depth studies are needed to explore these barriers further. Moreover, it does not allow for the identification of specific barriers that may have prevented individuals from brushing their teeth. Therefore, in-depth studies are needed to explore such barriers in detail. Also, we did not evaluate the effect of toothbrushing behaviors on actual dental caries experience. Although changes in toothbrushing frequency and timing were identified, the study design did not allow us to determine whether these behavioral changes translated into differences in caries prevalence or incidence. Third, as this study employed a cross-sectional design, caution is warranted when interpreting the findings in terms of causality. In addition, because the data were collected at a single point in time, temporal relationships between exposures and outcomes could not be established. Changes in behaviors or health status over time were not captured, and the potential influence of unmeasured confounding factors may remain. Fourth, this study utilized nationally representative data with a complex sampling design, incorporating stratification, clustering, and weighting in the analysis. However, due to the inherent characteristics of complex survey designs, there are limitations in estimating frequencies and standard errors compared to simple random sampling. Therefore, these methodological constraints should be carefully considered in the interpretation of the results.

Nevertheless, this study is meaningful in that it utilizes nationally representative data to compare toothbrushing practices before, during, and after the COVID-19 pandemic, stratified by age group. Future research should incorporate in-depth data collection to address potential barriers, such as school and family environments, social support, time constraints, and participation in oral health programs. These findings highlight the urgent need to develop safe and effective oral hygiene management strategies that can be implemented even during pandemics, supported by solid scientific evidence. Such efforts will be essential in establishing sustainable oral health care systems capable of withstanding future public health crises.

## Conclusions

The COVID-19 pandemic significantly impacted toothbrushing habits among school-aged children in South Korea. The decrease in brushing after lunch highlights the disruption of school-based oral hygiene programs, while the increase in brushing before bedtime suggests an adaptation to home-based routines. These findings emphasize the need to reinforce school-based oral health programs, ensuring resilience during public health crises. Future strategies should prioritize the sustainability of effective school-based oral hygiene education while ensuring adaptability to potential disruptions caused by pandemics or other public health crises.

## Data Availability

No datasets were generated or analysed during the current study.
